# Whole-genome sequencing reveals the effect of vaccination on the evolution of *Bordetella pertussis*

**DOI:** 10.1038/srep12888

**Published:** 2015-08-18

**Authors:** Yinghua Xu, Bin Liu, Kirsi Gröndahl-Yli-Hannuksila, Yajun Tan, Lu Feng, Teemu Kallonen, Lichan Wang, Ding Peng, Qiushui He, Lei Wang, Shumin Zhang

**Affiliations:** 1 Key Laboratory of the Ministry of Health for Research on Quality and Standardization of Biotech Products, National Institutes of Food and Drug Control, Beijing 100050, P. R. China; 2 TEDA School of Biological Sciences and Biotechnology, Nankai University, Tianjin 300457, P.R. China; 3 Department of Medical Microbiology and Immunology, Turku University, Turku 20520, Finland; 4 Key Laboratory of Molecular Microbiology and Technology, Ministry of Education, 23 Hongda Street, Tianjin 300457, P. R. China; 5 Department of Infectious Disease Surveillance and Control, National Institute for Health and Welfare, Turku 20520, Finland; 6 Department of Medical Microbiology, Capital Medical University, Beijing 100069, P. R. China; 7 State Key Laboratory of Medicinal Chemical Biology, Nankai University 300457, Tianjin, P. R. China

## Abstract

Herd immunity can potentially induce a change of circulating viruses. However, it remains largely unknown that how bacterial pathogens adapt to vaccination. In this study, *Bordetella pertussis*, the causative agent of whooping cough, was selected as an example to explore possible effect of vaccination on the bacterial pathogen. We sequenced and analysed the complete genomes of 40 *B. pertussis* strains from Finland and China, as well as 11 previously sequenced strains from the Netherlands, where different vaccination strategies have been used over the past 50 years. The results showed that the molecular clock moved at different rates in these countries and in distinct periods, which suggested that evolution of the *B. pertussis* population was closely associated with the country vaccination coverage. Comparative whole-genome analyses indicated that evolution in this human-restricted pathogen was mainly characterised by ongoing genetic shift and gene loss. Furthermore, 116 SNPs were specifically detected in currently circulating *ptxP3*-containing strains. The finding might explain the successful emergence of this lineage and its spread worldwide. Collectively, our results suggest that the immune pressure of vaccination is one major driving force for the evolution of *B. pertussis*, which facilitates further exploration of the pathogenicity of *B. pertussis*.

The 20^th^ century witnessed enormous successes in vaccination campaigns against infectious diseases, drastically reducing burden of these diseases. However, certain recent studies have reported that the effectiveness of viral vaccines, such as the influenza vaccine, has been affected by mismatches between vaccine components and the circulating virus due to evolutionary drift^1^,^2^. In the case of viral pathogens, herd immunity is considered to potentially contribute to evolutionary drift^1^,^3^, but it is unclear that how the bacterial pathogens adapt to vaccination due to bacteria’s slower rate of evolution compared with that of viruses.

*Bordetella pertussis* could serve as a favourable example for illustrating the adaptation of bacterial pathogens. Before the introduction of mass vaccination, pertussis was one of the primary causes of infant mortality, but since the introduction of whole-cell pertussis vaccines (WCVs) in many countries in the 1940s–1960s, the morbidity and mortality of pertussis have been declined dramatically^4^. However, since 1990s, pertussis resurgence has been observed in developed countries that have attained high vaccination coverage such as the Netherland, the United Kingdom and the United States^4^,^5^,^6^.

Although *B. pertussis* is a monomorphic pathogen, the most common antigenic alleles among current circulating strains, such as pertussis toxin A subunit (*ptxA*) 1 and pertactin (*prn*) 2, have been shown to differ from the alleles of the vaccine strains (*ptxA2 or ptxA3* and *prn1*) used in several countries in which vaccination has been implemented since the 1940s–1960s^7^,^8^,^9^,^10^, suggesting that pathogen adaptation may play a role in the emergence of pertussis^5^,^10^,^11^,^12^,^13^,^14^,^15^. Furthermore, a novel allele of pertussis toxin promoter (*ptxP*) 3 emerged in the 1990s, and has become prevalent in developed countries^15^,^16^,^17^,^18^. Compared with *ptxP1* strain, the *ptxP3* can produce more Ptx, which results in increased bacterial virulence^19^; this suggests that the expansion of clones carrying *ptxP3* might be associated with pertussis resurgence in the Netherlands and Australia^16^,^19^.

Contrasting the observations from developed countries with long histories of high vaccine coverage, in China, the *prn* 2 allele was first detected in 2000^11^, and its frequency was found to be only 16% during the 1997–2005 period. Furthermore, many of the pulsed-filed gel electrophoresis (PFGE) profiles of the examined Chinese strains belonged to the three lineage clusters (group I, II and III) that resembled most of the strains circulating in Europe before the 1990s^11^. In Senegal, where large-scale pertussis vaccinations with WCVs were started in the late 1980s and the coverage was generally low, the predominant strains also belong to these 3 groups^20^. These observations indicate that distinct immunization strategies may be linked to the microevolution of *B. pertussis*.

Recently, a comparative genomic study based on a global collection of 343 *B. pertussis* isolates revealed that the worldwide *B. pertussis* population underwent major changes during last 60 years; the phylo-geographic analysis of these strains suggests that adaptive evolution of this pathogen is closely associated with vaccine introduction and that new emerged strains spread rapidly between countries^21^. However, several questions remain unanswered, including these: (1) Why do the types of isolates prevalent before the introduction of vaccination still predominate in areas of low vaccination coverage? (2) Do the evolution rates of *B. pertussis* population in certain countries with high vaccination coverage differ from the rates in countries in which the coverage is low? To further understand how vaccination affects *B. pertussis* evolution and obtain insight into how this pathogen has adapted to immune pressure from vaccination, we performed whole-genome sequencing to characterise 40 isolates from 1956 to 2008 in Finland, a country with high vaccination coverage and China, a country with relatively low coverage. The results were also compared with the sequences of 11 previously published strains from the Netherlands^7^,^22^, where pertussis vaccination was introduced in 1953.

## Results

### SNPs and phylogenetic analyses

The sequence reads obtained for each isolate were assembled in the genomes, which resulted on average in 105-fold coverage per genome (Supplementary Table S1). These sequences, combined with 11 previously published whole-genome sequences of Dutch isolates, were mapped against the complete chromosome of the *B. pertussis* strain CS. Our analysis focused on the non-repetitive components of each genome. Repetitive sequences, including variable-number tandem-repeat sequences and 3 types of insertion sequences (*IS*), *IS* 481 (239 copies), *IS* 1002 (6 copies) and *IS* 1663 (17 copies), accounted for 6.3% of genome of the strain CS^23^. After excluding these repetitive sequences, 825 SNP sites were identified in the non-repetitive genome (Supplementary Tables 2 and 3). Further analysis revealed that 120 of these SNPs appeared in high-density clusters, indicating that they might have originated from recent recombinant events^24^; 101 SNPs in *B. pertussis* isolates from recent periods (since 1990s) (Supplementary Table S3). Moreover, although 50.8% (61/120) of the SNPs were located at non-coding regions and genes of unknown function, it was noted that certain recombination events had occurred and included genetic determinants for recognised surface antigens and two-component regulatory systems, such as genes encoding Ptx, filamentous hemagglutinin (FHA) and two-component histidine kinase, which modulated host-cell interactions.

**Figure 1:**
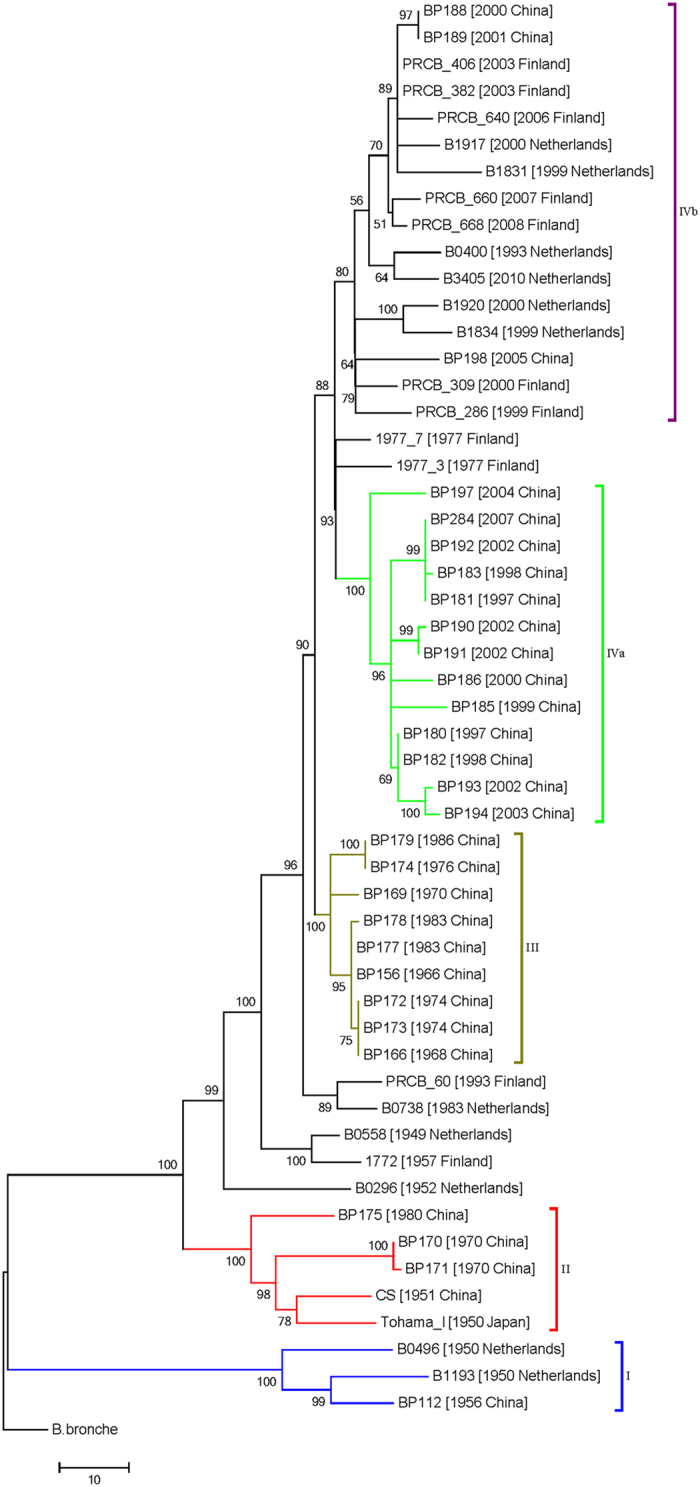
Maximum likelihood phylogenetic tree for *B. pertussis* based on SNPs across the entire core genome, excluding probable recombination events. *B. bronchiseptica* was used as the outgroup to root the tree. The evolutionary relationships were inferred using the neighbor-joining method. The percentages of replicate trees in which the associated taxa clustered together based on the bootstrap test (100 replicates) are presented next to the branches.

**Figure 2:**
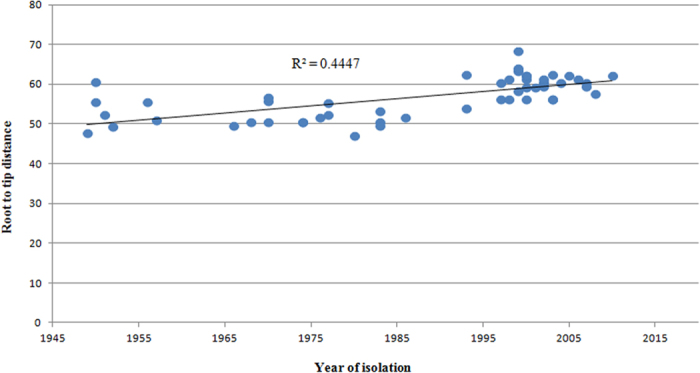
Linear regression plot of *B. pertussis* displaying the correlation (R^2^) between the root-to-tip distance (y-axis) and the date of isolation (x-axis).

We excluded the aforementioned SNPs from the phylogenetic analyses because the recombination masked the true phylogenetic signal. Using *B. bronchiseptica* as the outgroup, a maximum likelihood phylogenetic tree that also included the published genome of *B. pertussis* Tohama I^25^ was constructed based on the remaining 705 SNPs (Fig. 1). Detailed information regarding these SNPs was provided in Supplementary Table S2. We also constructed a phylogenetic tree with roots by using the *B. pertussis* typing strain 18323 (Supplementary Fig. S1). The two trees exhibited the same topology, further supporting the hypothesis that strain 18323 is highly phylogenetically distinct from other *B. pertussis* strains, and might have been derived through a unique evolutionary process from the most recent common ancestor (MRCA)^21^,^26^. The resulting phylogeny recapitulated the 4 major groups previously as identified by the PFGE analyses^11^,^12^ but provided more accurate estimates of branch lengths and substantially increased the resolution. The strains from different countries within the same period (pre- or post- vaccination era) were clustered closely in the phylogenetic tree. Furthermore, the strains within each group were separated by up to 300 SNPs, and the strains of clade IV appeared to be the most diverse. 35 SNPs shared by all members of group III and IV discriminated them from the isolates in other groups. Lastly, clade IV contained most of the strains (88.6%, 31/35) from 1977 to 2008, and this clade was divided into 2 subgroups, IVa and IVb, separated by 121 SNPs. The results indicated that the number of SNPs from the root varied between the different clades and that the root-to-tip distances of individual isolates correlated with their date of isolation (*R*^2^ = 0.44; Fig. 2), suggesting that *B. pertussis* adapted through successive SNP accumulation over time^7^,^21^. Furthermore, the correlation between the year of isolation and the root-to-tip distances of individual Chinese strains (*R*^2^ = 0.58) differed from those of Finnish and Dutch strains (*R*^2^ = 0.35; Supplementary Fig. S2), indicating that SNP accumulation rates over time might vary between different countries.

### Variations in mutation rate

Single-copy genes are conserved genetic markers and are widely used for phylogenetic analyses of numerous species^27^,^28^,^29^. To investigate the potential cause of the aforementioned heterogeneity in genomic diversity, we performed a BEAST analysis on the single-copy genes in the genomes of isolates from different periods and countries. Our results demonstrated that the substitution rates of the isolates from period 2 were 6.0-fold higher than those of isolates from period 1 [substitutions per site per year: 4.60 × 10^−5^, 95% confidence interval (CI): 2.03–7.78 × 10^−5^ versus 7.64 × 10^−6^, 95% CI: 3.03 × 10^−10^ to 1.63 × 10^−5^] (Supplementary Fig. S3). Furthermore, the substitution rates of the Finnish and Dutch *B. pertussis* populations were higher than those of the Chinese population (substitutions per site per year: 1.59 × 10^−5^, 95% CI: 3.98 × 10^−6^ to 2.87 × 10^−5^ versus 3.06 × 10^−6^, 95% CI: 1.25–5.18 × 10^−6^) (Supplementary Fig. S3). Similar results were also obtained in the BEAST analysis of SNPs in the *B. pertussis* population (Supplementary Fig. S4). These results suggested that the substitution mutation rate differed substantially between isolates from different countries or periods.

### Potential signals of selection

Our results identified 307 nonsynonymous SNPs, which represented 43.5% of all SNPs found here (Supplementary Table S2 and Supplementary Fig. S5). Further analysis showed that the nonsynonymous SNP densities of isolates from clades IVa and IVb were higher than those of isolates from clade I and II (*P* < 0.01). It is generally accepted that homoplasies are traits that species share due to convergent evolution that constitute a dependable signal of selective pressure. Here, 42 homoplastic SNPs were identified, of which 27 occurred within the clade III and IV lineages (Supplementary Table S4); 16 of these 27 SNPs were located in coding regions, 11 were intergenic. Intriguingly, one SNP were present at between the *bvgA*-encoded virulence factors transcription regulator and the *fhaB* encoded adhesion protein FHA. Furthermore, 11 of the homoplastic SNPs associated with coding genes were nonsynonymous SNPs. Although these coding genes containing homoplasies are poorly characterised, a few of the representative genes are of interest, such as *sphB1* and *bscl*, which are under the control of the *Bordetella* virulence regulon (*bvg*). The product of *sphB1* was reported to be an outer membrane protein, associated with host-cell adhesion and invasion of *B. pertussis*^30^,^31^. The gene *bscl* identified in *B. bronchiseptica* and *B. pertussis* encodes a type III secretion protein that is recognised to be essential for bacterial pathogenicity^23^,^25^,^32^. Another *bvg*-regulated virulence gene, *ptxc* which encodes Ptx subunit 3, also contained a homoplastic SNP. Collectively, these data indicated that adaptive bacterial evolution might contribute to the substitution-rate differences.

### Genomic deletions and ongoing gene loss

To further investigate the evolution of distinct lineages, we analysed the nucleotide changes that induced gene disruption and genome size reduction. The clade II strain CS, which was isolated in Beijing, China, in 1951, was used as a reference to compare the genome sequences of the examined isolates. In addition to base substitutions, genomic deletions were common in the sequenced isolates, but insertions were infrequent. The mean deletion size largely exceeded the mean insertion size: only 732 bp were gained, but 19.4 kb were lost (Supplementary Fig. S6). The deletions ranged in size from 1 bp to 6.4 kb, and most of the deleted regions included protein-coding sequences, resulting in partial or complete deletion of *B. pertussis* genes (Supplementary Fig. S6). Deletions, insertions and nucleotide substitutions affect the corresponding gene. However, their effects on protein function cannot be readily to predict; thus we focused on genes that were completely deleted. Of the 150 deleted genes found in various lineages (Supplementary Table S5), a few corresponded to variant regions previously identified based on DNA microarray analyses^33^. Several of these genes are involved in replication, recombination and repair, transcription and unknown functions. When the deletions were mapped to the phylogenetic tree, these lineages were found to display gene loss over time (Supplementary Figs S6 and S7), further supporting the ongoing reductive evolution in the global *B. pertussis* population^21^.

In addition to identifying the aforementioned genes affected by deletion events, we detected 9 nonsense SNPs that have occurred since the MRCA. These SNPs introduce stop codons into protein-coding genes and terminate translation (Supplementary Table S2). The published strains Tohama and CS both contain more than 300 pseudogenes^23^,^25^. Pseudogenes constitute 9.4% of all *B. pertussis* genes, a considerably higher proportion than that in other *Bordetella* species, such as *B. bronchiseptica* (0.4%), *B. parapertussis* (5.0%), *B. avium* (2.0%) and *B. petrii* (2.5%)^25^,^34^,^35^.

### Emergence of the *ptxP3* lineage

**Figure 3:**
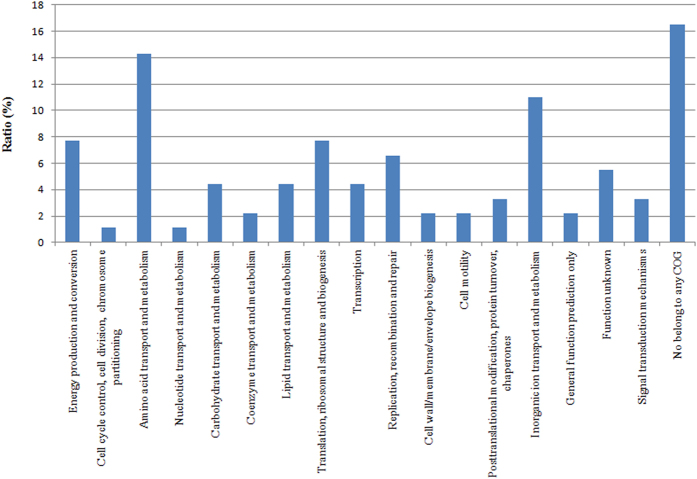
Clusters of orthologous genes associated with the specific SNPs in the *ptxP3* strains.

Almost all subgroup IVb isolates contained the novel *ptxP3* allele, whereas subgroup IVa isolates contained *ptxP1*. The phylogenetic tree clearly revealed that these 2 subgroups shared a common ancestor. The results of Bayesian phylogenetic analysis suggested that these lineages diverged more than 20 years ago, which agrees with the earliest reports of *ptxP3* strains in the 1990s^19^,^36^. We also examined the distribution of SNPs in the *ptxP3* lineage, and identified 116 specific SNPs, of which 54 and 10 SNPs were distributed in our Finnish and Chinese isolates, respectively (Supplementary Table S2). Besides the above 3 previously mentioned SNPs in coding regions (*ptxc*, *sphb1* and *bscl*), 85 other SNPs were located in coding regions and 28 SNPs were intergenic (located in non-coding regions). The SNPs in the coding regions corresponded to 17 different clusters of orthologous genes (COGs). The highest COG frequencies were related to amino acid transport and metabolism (14.4%), and inorganic ion transport and metabolism (11.1%) (Fig. 3). Furthermore, one nonsynonymous SNP was located in *fim3* and caused an alanine (A) to glutamic acid (E) substitution. Fimbriae 3 (Fim3), also known as pili or agglutinogen, is expressed on the surface of *B. pertussis* and is considered to be involved in the bacterial attachment to the respiratory tract^37^.

## Discussion

Isolate selection is critical for comparative analyses because it uncovers mutations that differ between the examined isolates. The 29 Chinese and 11 Finnish isolates studies here were sampled from reliable phylogenies based on the molecular genotyping of >500 strains from Finland and >100 strains from China^11^,^12^,^38^ to provide a satisfactory representation of the genetic diversity of *B. pertussis* in these two countries. To capture most of the variation in *B. pertussis* isolates from different countries at the whole-genome level and to further understand how vaccination affects bacterial evolution, 11 previously published genomes of *B. pertussis* isolates obtained between 1949 and 2010 from the Netherlands^7^,^22^ were also included in this study.

Vaccinations against pertussis have been introduced since 1950s in many countries. The initial WCVs were replaced by less reactogenic acellular pertussis vaccines (ACVs) in the 1990s in developed countries^4^. Many studies have demonstrated the marked changes in the *B. pertussis* populations in these countries following the introduction of ACVs^11^,^17^,^18^,^21^. *B. pertussis* strains from different countries with similar vaccination histories are genetically similar^22^,^39^,^40^,^41^. Our studies also revealed that Finnish and Chinese *B. pertussis* isolates from similar period were phylogenetically closely related, further supporting the hypothesis that *B. pertussis* strains do not exhibit geographic specificity^21^,^33^. Although the exact cause for this is unknown, it might be associated with recent population migration, human admixture, and strain transmission.

*B. pertussis* is an obligate human pathogen, and evolutionary pressure is likely dominantly mediated by the host immune response^9^. Comparative genomic analysis has provided evidence that the world-wide *B. pertussis* population is evolving in response to vaccine introduction^21^. Extending previous findings^11^,^21^, our results showed that the molecular clock moved at a dissimilar rate in different countries and periods, which might have resulted in the large heterogeneity in the number of SNPs that accumulated over time. According to WHO estimates, the rate of vaccination coverage was only 58% in 1983 in China, as compared with 94% and 97% in Finland and the Netherlands, respectively^42^. A variation in vaccination coverage might exert different selective pressures on *B. pertussis* populations^11^,^20^. In Finland and the Netherlands, ACVs were introduced in 2005, whereas in China, WCVs were not completely replaced by ACVs until 2012. For understanding the evolution of *B. pertussis*, evaluating the relative impact of ACVs and WCVs is critical. However, we could not analyse this impact because our study included only 3 Finnish strains collected after ACV introduction. Recently, Sealey *et al* have demonstrated that in the ACV era, genes encoding ACV antigens are evolving more rapidly than other cell surface proteins of *B. pertussis* isolates^43^. Collectively, these results provide direct evidence that *B. pertussis* evolution has accelerated since the introduction of vaccination. This evolution might also explain why isolates belonging to the PFGE group III, which predominated before the introduction of ACVs in industrialised countries, are still prevalent in areas with low vaccination coverage, and thus contribute to the differences observed in the frequency of *prn2* alleles between these countries^11^,^20^.

In agreement with previous studies^21^,^43^, an over abundance of nonsynonymous and homoplastic SNPs was identified in *B. pertussis* isolates from both China and Finland, which provided additional evidence for adaptive selection in *B. pertussis* strains from the post-vaccination era. However, most of the detected homoplasies were located in modern isolates, which were primarily categorised into clades III and IV, indicating that convergent evolution might occur over short periods. Although the precise effects of these homoplasies in *B. pertussis* genes are unknown, we found 3 homoplastic SNPs in 3 genes (*ptxc, sphB1* and *bscl*) likely associated with bacterial virulence under the control of *bvg*, which consisted with 33% of the homoplastic SNPs located in bvg-activated genes in global phylogenic analysis of *B. pertussis*^21^. Notably, one homoplasic SNP in *bscl* was identified in *B. pertussis* strains collected in different countries^21^. The *bscl* encodes a component of the *Bordetella* type III secretion system^32^, which subverts innate and adaptive immune responses during *B. pertussis* infection and promotes bacterial persistence^44^. Recently, Han *et al* have reported that the type III effector BteA is expressed at higher levels in *B. pertussis* non-vaccine type strains than in vaccine-type strains, which suggests that increased BteA protein expression might play a key role in the evolution of *B. pertussis*^45^. Intergenic SNPs have been suggested to be capable of altering gene expression level^46^. Furthermore, an abundance of homoplasic SNPs in intergenic region might affect the transcription of related genes. Although the functions of most of the identified genes are unknown, we suspect that the intergenic SNP located at between *bvgA* and *fhaB* might be associated with pathogen adaptation. Taken together, the adaptive mutations detected in surface antigenic and *bvg*-controlled genes further emphasize the strong selective pressure on the *B. pertussis* genome due to herd immunity^21^.

Our whole-genome comparison provided insights into the spectrum of genetic variation in the *B. pertussis* population. In contrast to the accumulation of SNP mutations over time, numerous deletions were identified in different lineage and continuous gene loss was observed. Although a high coverage of bacterial genomes was obtained using the Illumina sequencing platform and *de novo* assemblies were performed, a few gaps might remain between contigs due to the limitation of unfinished genomes^47^. Therefore, to reduce error by increasing alignment accuracy, we only analysed insertions or deletion flanked by at least 20 bp of gapless matches. Similar to other genetically monomorphic bacterial pathogens, such as *Yersinia pestis*^48^, a remarkably high number of transposed sequences, IS elements and pseudogenes are present in the *B. pertussis* genomes compared with other *Bordetella* species^25^,^34^,^35^. Indeed, IS elements have been reported to play a major role on causing gene loss in *B. pertussis* population, which benefitted from the increased fitness^21^,^33^. Furthermore, our whole-genome analysis also revealed an ongoing formation of pseudogenes in *B. pertussis*. High pseudogene frequencies are associated with host restriction in a variety of bacterial species^49^. This phenomenon is potentially caused by high rates of mutational fixation resulting from the accelerated genetic shift due to evolutionary bottlenecks associated with host adaptation and a reduction in the effective population size. Collectively, these data further support the hypothesis that evolution in this host-restricted pathogen might be dominated by genetic shift and gene loss rather than by the acquisition of new genes^21^,^33^,^43^. On the other hands, previous studies have also reported that rearrangements mediated by IS elements contribute to the genome reduction in *B. pertussis* when it evolved from a *B. bronchiseptica* like ancestor^25^. Chromosomal rearrangements can even occur during subculturing of *B. pertussis in vitro*^50^. Altogether, rearrangement might also serve as a key mechanism in *Bordetella* evolution.

Recent studies also demonstrated that *ptxP3* is the dominant allele in clinical isolates from many countries with high vaccine coverage^15^,^16^,^36^. In this study, we showed that *ptxP3* was associated with the emergence of sub-branch that contained the currently circulating isolates from different countries. Mooi *et al* reported that the *ptxP3* strains can lead to the increased production of Ptx than the *ptxP1*, resulting in increased virulence and immune suppression^19^. Aside from the SNP mutations in the *ptxP* promoter, our whole-genome analysis identified 116 specific SNPs in the *ptxP3* lineage. These polymorphisms might facilitate the adaptability of the *ptxP3* strains. Notably, 3 SNPs specific to British *ptxP3* strains^43^ were also identified in this present study. Collectively, these results highlight that the SNPs identified among *ptxP3* strains will serve as potential genotypic markers in phylogenetic genotyping studies, particularly in the monitoring of *ptxP3* strains worldwide. A recent genome-wide association study have also demonstrated that compared to *ptxP1* strains, the *ptxP3* strains express higher levels of a series of genes belonging to amino acid biosynthesis, energy metabolism, regulation and central/intermediary metabolism categories, and that wild-type *ptxP3* strains are relatively more effective colonizers in an animal infection model^51^. These characteristics might have caused the recent emergence of the *ptxP3* lineage and its worldwide spread.

In conclusion, our whole-genome comparative analysis supports the hypothesis that the evolution of *B. pertussis* has been dominated by ongoing genetic shift and gene loss^21^,^33^,^43^. Furthermore, numerous SNPs specific to the *ptxP3*-containing strains were identified here, which might explain the recent emergence and worldwide spread of this successful lineage. More importantly, we found that the molecular clock moved at different rates in different countries and periods, suggesting that the evolution of the *B. pertussis* population is closely associated with the extent of vaccination coverage. These results provide new and crucial evidence that the immune pressure from vaccination is one major driving force for the evolution of *B. pertussis*. We expect these data to enrich the understanding of evolutionary mechanisms in pathogens in the presence of different vaccination coverage levels. The genomic data presented here will also facilitate further exploration of the pathogenicity of *B. pertussis*.

## Methods

### Bacterial strains

We selected 29 Chinese and 11 Finnish isolates from 1956 to 2008 based on molecular genotyping in accordance with their occurrence frequencies^11^,^38^. The selected strains were representative of the most prevalent PFGE profiles and genotypes during each period (Table 1 and Supplementary Table S1). Furthermore, *B. pertussis* strain CS, which isolated in China in 1951 and is widely used as a vaccine strain for ACV production in China, was included in the analysis^23^. All isolates were cultured on Bordet-Gengou agar supplemented with 15% sheep blood for 72 h at 37 °C, and genomic DNA was isolated using the Promega Wizard® Genomic DNA Purification Kit (Promega, Madison, WI, USA) according to the manufacturer’s instructions.

To comprehensively investigate how vaccination affects the evolution of bacterial genomes, 11 published genomes of Dutch isolates from 1949 to 2010 were included in the phylogenetic analysis of *B. pertussis*. These isolates were separated into 2 periods based on the time of WCV introduction and the vaccine coverage in each country (Table 1). Period 1 comprised the period before the onset of mass vaccination using WCVs and the early post-vaccination period that resulted in a low coverage rate, including 10 Chinese isolates from 1951 to 1977 and 5 European isolates (1 Finnish, 4 Dutch) from 1949 to 1957. Period 2 covered the post-vaccination period, including 20 Chinese isolates from 1980 to 2007 and 17 European isolates (10 Finnish, 7 Dutch) from 1977 to 2010 (Supplementary Table S1).

### Genome sequencing and comparative genomics

Whole-genome sequencing of 40 isolates was performed using the Solexa Genome Analyzer IIx 75-bp paired-end platform (Illumina, Little Chesterford, UK). The Illumina data were *de novo* assembled using Velvet, and multi-contig draft genomes were generated for each isolate. Details for each genomic assembly were summarized in Supplementary Table S1. Open reading frames of at least 30 amino acids in length were predicted using Glimmer 3.0 and were manually verified according to the annotation of *B. pertussis* CS^23^. The transfer RNA and ribosomal RNA genes were predicted using tRNAscan-SE. Artemis was used to collate the data and facilitate the annotation^52^. Functional predictions were based on BLASTp similarity searches using the UniProtKB, GenBank, and Swiss-Prot protein databases. Genome comparisons were performed to search for insertion and deletion events. Blocks of sequences that were substantially shared by *B. pertussis* CS and each isolate were identified using BLAST, and the alignment within each block was based on the Mauve method^53^ using a seed length of 15. In order to reduce error by improving alignment accuracy, only insertions or deletions, flanked by at least 20 bp of gapless matches were considered to be indel events^54^.

### Detection of SNPs and phylogenetic analysis

The generated Solexa reads were called from duplicates containing identical reads that were presumably derived from replicates during the PCR step. Then, the reads were mapped to the genome of *B. pertussis* CS (accession number NC_017223) using BWA^55^ under the default parameters, which allow for 3% mismatches. All reads of extremely large or small insert size (<50% or >200% of normal) were remapped by using BLASTn at an e-value of 0.0001 and using the -F F flag. We only considered read pairs that mapped at an appropriate separation and contained at least one mapped end in a non-repeat region. The SAMtools program was used to calculate the per-position coverage and the base calls for each position^56^. The SNPs in the sequenced strain were called if the position was covered by at least 10 reads and at least 80% of the covering reads displayed the same mismatch^57^. SNP clusters within the recombination region were excluded as described previously^24^, and SNPs from regions that were not present in all genomes were also excluded. A SNP was considered homoplastic if the allele pattern did not agree with the tree topology. A phylogeny was constructed based on the SNPs using RAxML, which was based on the root of *B. bronchiseptica* or *B. pertussis* strain 18323. A regression analysis was conducted on the root-to-tip genetic distances against the sampling date according to a neighbor-joining tree using the program Path-O-Gen V1.4 (http://tree.bio.ed.ac.uk/software/pathogen/). The SNP densities were calculated by determining the number of SNPs per bp of each gene, and the differences between distinct clades were analysed using the nonparametric Wilcoxon test of SPSS 20.0 software (SPSS Inc., Chicago, IL, USA). *P* < 0.05 was considered statistically significant.

To comprehensively investigate the evolution sites in *B. pertussis* strains from different countries and periods, the SNPs and single-copy orthologous clusters identified in this study were utilized to estimate the nucleotide substitution rate of each site (substitutions per site per year) using the BEAST package^58^. BEAST analysis can incorporate the date of isolates into the model and provide a source of information on the overall rate of evolutionary change when the differences in the dates are associated with the tips of the phylogenetic tree. The single-copy orthologous gene families were identified as previously described^28^,^29^. Briefly, we compared and clustered the complete genomes of all *B. pertussis* strains in this study using the software OrthoMCL^29^, identified 179 single-copy orthologous families shared among all genomes. We used the jModelTest program^59^ to select the best-fitting substitution model for our sequences according to the Bayesian information criterion, and the date of isolation of each isolate was used to calibrate the tree, and finally these analyses were performed using a log-normal relaxed molecular clock, which allows for different mutation rates on different branches. Each run (4 in total) spanned 100 million generations, and the model parameter values and trees were sampled every 10,000 generations. The results were visualised using Tracer v1.5 to assess convergence.

## Additional Information

**How to cite this article**: Xu, Y. *et al*. Whole-genome sequencing reveals the effect of vaccination on the evolution of *Bordetella pertussis*. *Sci. Rep*. **5**, 12888; doi: 10.1038/srep12888 (2015).

## Supplementary Material

Supplementary Information

Supplementary Table S1

Supplementary Table S2

Supplementary Table S3

Supplementary Table S4

Supplementary Table S5

## Figures and Tables

**Table 1:** Pertussis vaccination program of the three countries.

Country	Start	Vaccination schedule
Finland	1952	**Primary:** DTwP at 3,4,5 months;**Boosters:** DTwP at 20–24 months
	2003	**Primary**: DTwP at 3,4,5 months;**Boosters**: DTwP at 20–24 months and dTap at 6 years
	2005	**Primary**: DTaP at 3,5,12 months;**Boosters**: dTap at 4 and 14 years
China*	1960s	**Primary**: DTwP at 3,4,5 months;
	1978	**Primary**: DTwP at 3,4,5 months;**Boosters**: DTwP at 18–24 months
	2007	**Primary**: DTwP or DTaP at 3,4,5 months;**Boosters**: DTwP or DTaP at 18–24 months
Netherlands	1953	**Primary**: DTwP at 3,4,5 months;**Boosters**: DTwP at 4 years
	1962	**Primary**: DTwP-IPV at 3,4,5, 11 months
	1993	**Primary**: DTwP-IPV-Hib at 3,4,5, 11 months
	1999	**Primary**: DTwP-IPV-Hib at 2,3,4, 11 months
	2001	**Primary**: DTwP-IPV-Hib at 2,3,4, 11 months;**Booster**: DTaP at 4 years
	2005	**Primary**: DTaP-IPV-Hib at 2,3,4, 11 months;**Booster**: DTaP at 4 years
	2006	**Primary**: DTaP-IPV-Hib at 2,3,4, 11 months;**Booster**: dTap at 4 years

Notes: *Although DTaP was phased in from the late 1990s, DTwP have been completely replaced by DTaP until 2012 in China. DTwP, diphtheria-tetanus-whole cell pertussis; DTaP, diphtheria-tetanus-acellular pertussis; dTap, diphtheria-tetanus-whole cell pertussis (with reduced content of pertussis antigens); IPV, inactivated polio virus; Hib, *haemophilus influenza* type b
