# Author Correction: Whole-genome sequencing reveals the effect of vaccination on the evolution of *Bordetella pertussis*

**DOI:** 10.1038/s41598-021-99283-z

**Published:** 2021-09-30

**Authors:** Yinghua Xu, Bin Liu, Kirsi Gröndahl-Yli-Hannuksela, Yajun Tan, Lu Feng, Teemu Kallonen, Lichan Wang, Ding Peng, Qiushui He, Lei Wang, Shumin Zhang

**Affiliations:** 1 Key Laboratory of the Ministry of Health for Research on Quality and Standardization of Biotech Products, National Institutes of Food and Drug Control, Beijing, 100050 P. R. China; 2 TEDA School of Biological Sciences and Biotechnology, Nankai University, Tianjin, 300457 P.R. China; 3 Department of Medical Microbiology and Immunology, Turku University, Turku, 20520 Finland; 4 Key Laboratory of Molecular Microbiology and Technology, Ministry of Education, 23 Hongda Street, Tianjin, 300457 P. R. China; 5 Department of Infectious Disease Surveillance and Control, National Institute for Health and Welfare, Turku, 20520 Finland; 6 Department of Medical Microbiology, Capital Medical University, Beijing, 100069 P. R. China; 7 State Key Laboratory of Medicinal Chemical Biology, Nankai University, 300457, Tianjin, P. R. China

Correction to: *Scientific Reports* https://doi.org/10.1038/srep12888, published online 18 August 2015

The original version of this Article contains an error in the spelling of the author Kirsi Gröndahl-Yli-Hannuksela which is incorrectly given as Kirsi Gröndahl-Yli-Hannuksila.

