# Supplementary material for: Whole-genome sequencing reveals the effect of vaccination on the evolution of Bordetella pertussis
Source: Sci Rep. 2015 Aug 18;5:12888. doi: 10.1038/srep12888 (PMC4539551; doi:10.1038/srep12888)
Supplement: Supplementary Information [file srep12888-s1.doc]

**Supplementary Information**

**Whole-genome sequencing reveals the effect of vaccination on the evolution of *Bordetella pertussis***

**Yinghua Xu^1＋^, Bin Liu^2,4＋^, Kirsi Gröndahl-Yli-Hannuksila^3＋^, Yajun Tan^1^, Lu Feng^2,4^, Teemu Kallonen^3^, Lichan Wang^1^, Ding Peng^2,4^, Qiushui He^3,5,6*^, Lei Wang^2,4,7*^, Shumin Zhang^1*^**

1. Key Laboratory of the Ministry of Health for Research on Quality and Standardization of Biotech Products, National Institutes of Food and Drug Control, Beijing 100050, P. R. China

2. TEDA School of Biological Sciences and Biotechnology, Nankai University, Tianjin 300457, P.R. China

3. Department of Medical Microbiology and Immunology, Turku University, Turku 20520, Finland

4. Key Laboratory of Molecular Microbiology and Technology, Ministry of Education, 23 Hongda Street, Tianjin 300457, P. R. China

5. Department of Infectious Disease Surveillance and Control, National Institute for Health and Welfare, Turku 20520, Finland

6. Department of Medical Microbiology, Capital Medical University, Beijing100069, P. R. China

7. State Key Laboratory of Medicinal Chemical Biology, Nankai University 300457, Tianjin, P. R. China

***Corresponding author:**

Dr. Qiushui He, Department of Medical Microbiology and Immunology, Turku University, Turku 20520, Finland; Tel: 358-2-3337429; Fax: 358-2-2330008; Email: Qiushui.He@utu.fi

Dr. Lei Wang, TEDA School of Biological Sciences and Biotechnology, Nankai University, 23 Hongda Street, TEDA, Tianjin 300457, People’s Republic of China. Tel: 86-22-66229588. Fax: 86-22-66229596. E-mail: wanglei@nankai.edu.cn

Dr. Shumin Zhang, National Institute for the Control of Pharmaceutical and Biological Products, Temple of Heaven, Beijing 100050, People's Republic of China; Tel: 86-10-67095598; Fax: 86-10-67051912; E-mail: [zhangsm@nifdc.org.cn](mailto:zhangsm@nifdc.org.cn)

**^＋^**These authors contributed equal to this work.

| **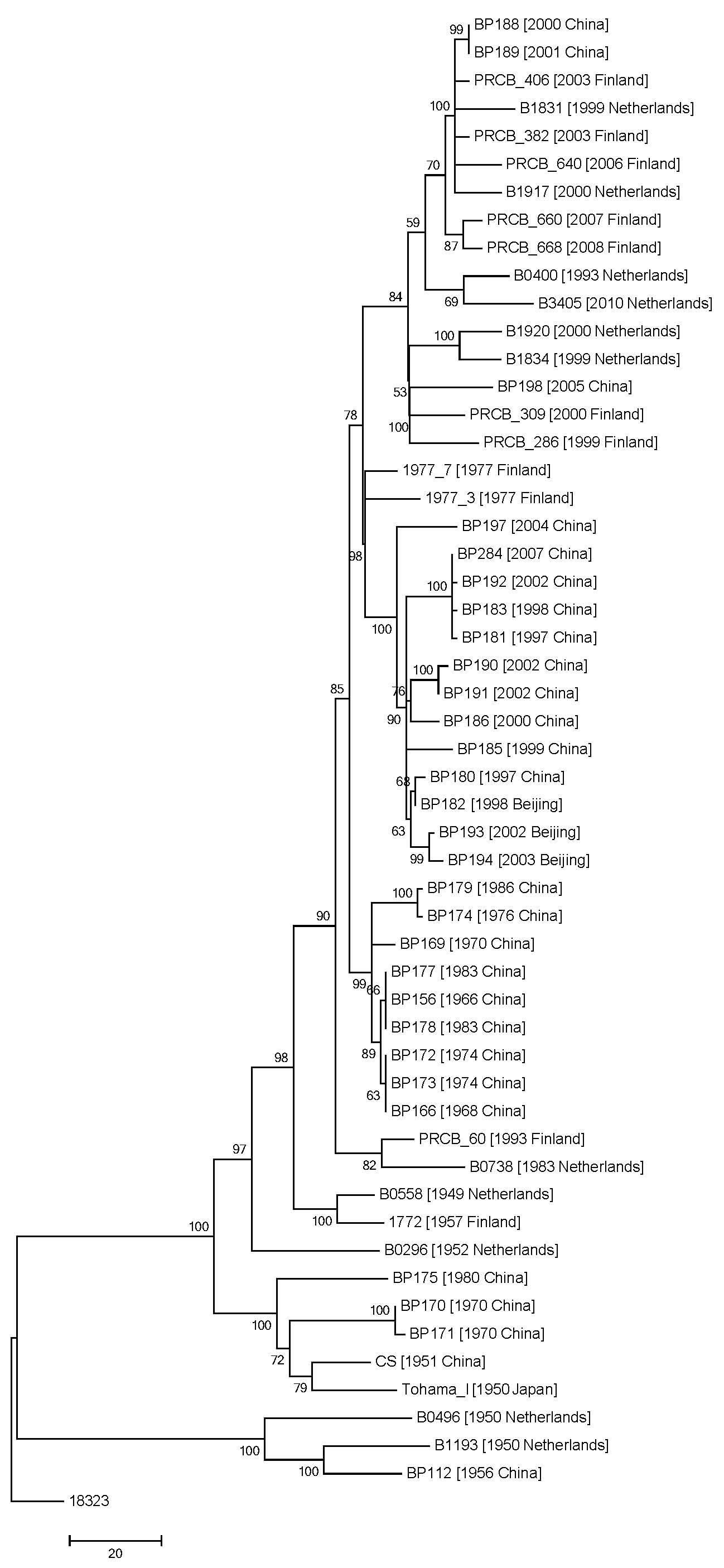** |
| --- |
| **Supplementary Figure S1.** Maximum likelihood phylogenetic tree of the *B. pertussis* isolates based on SNPs across the entire core genome, excluding probable recombination events. *B. pertussis* 18323 was used as the outgroup to root the tree. The evolutionary relationships were inferred using the neighbor-joining method. The percentages of replicate trees in which the associated taxa clustered together based on the bootstrap test (100 replicates) are presented next to the branches. |

| **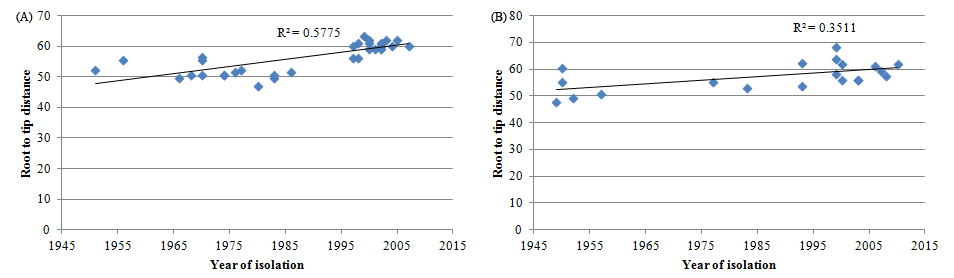** |
| --- |
| **Supplementary Figure S2**. Linear regression plot of *B. pertussis* displaying the correlation (R^2^) between the root-to-tip distance (y-axis) and the date of isolation (x-axis). (A) Strains from China. (B) Strains from Finland and the Netherlands. |

| 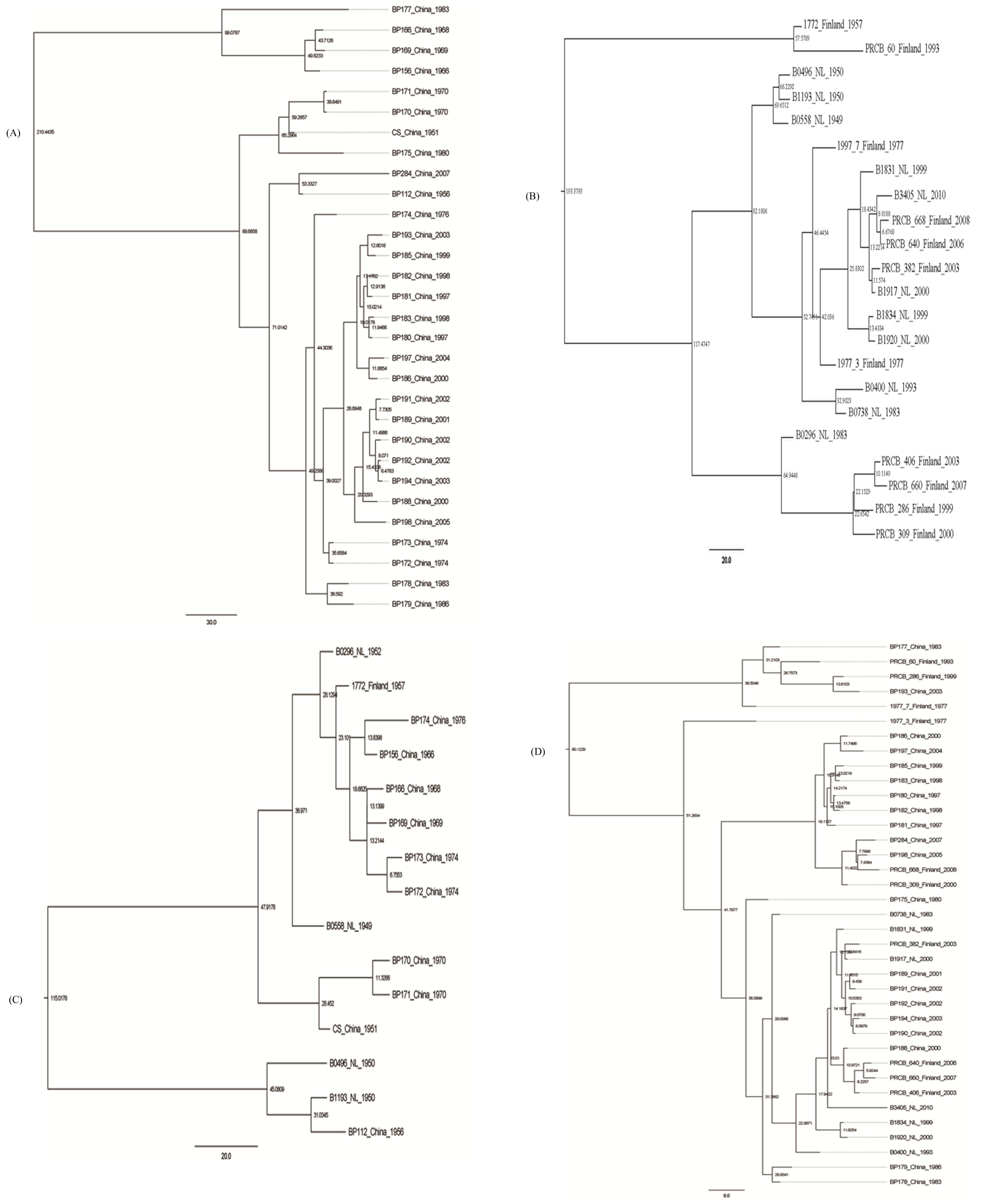 |
| --- |
| **Supplementary Figure S3**. A maximum clade credibility tree based on the single-copy orthologous families identified in the *B. pertussis* isolates from different countries and periods. (A) Strains from China. (B) Strains from Finland and the Netherlands. (C) Strains from period 1. (D) Strains from period 2. The numbers on each branch represent the time of divergence from the most recent common ancestor. |

| 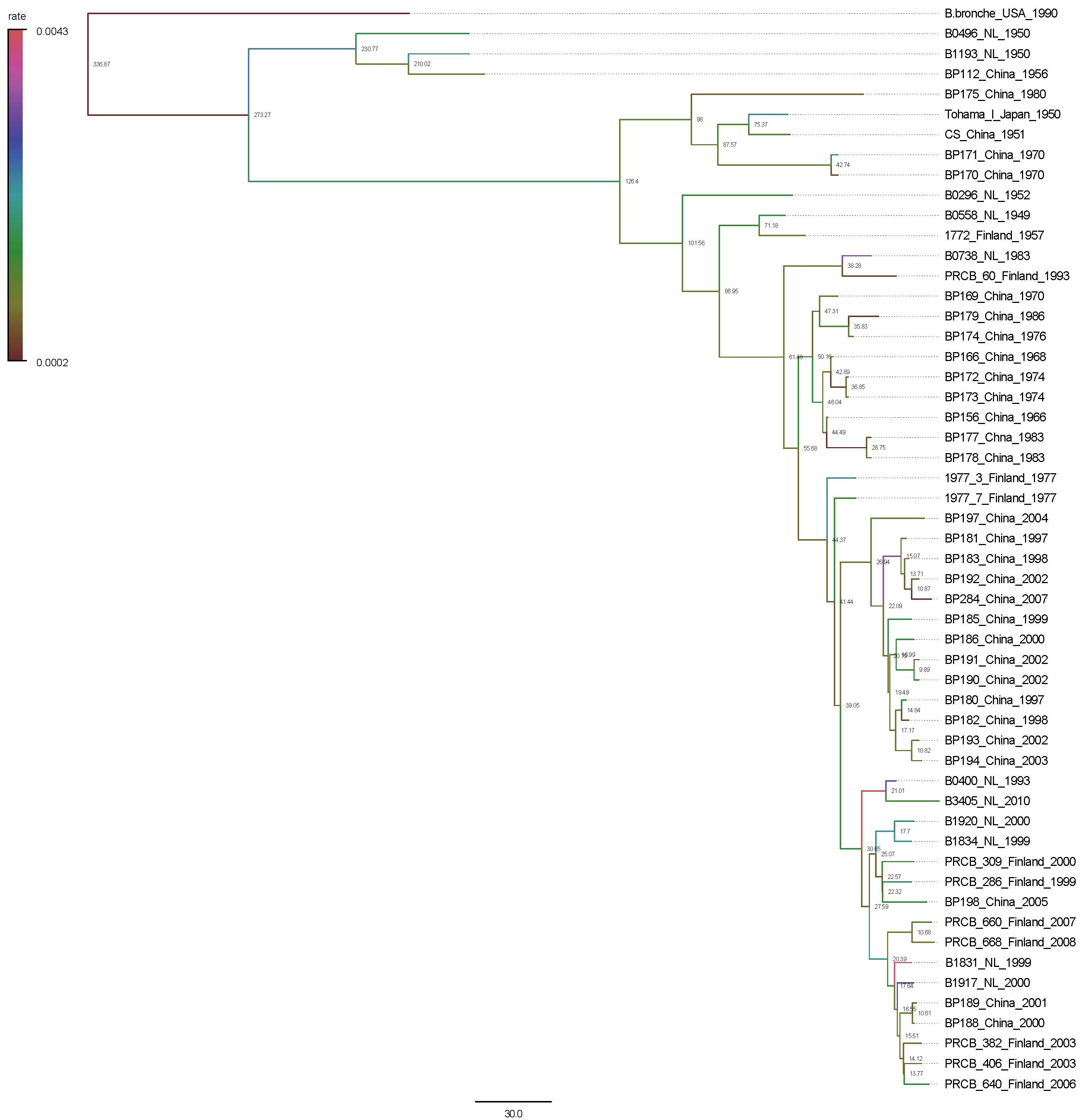 |
| --- |
| **Supplementary Figure S4.** A maximum clade credibility tree based on the SNP data identified in this study under a coalescent model using a relaxed molecular clock. The branch colors indicate the estimated branch substitution rates on a logarithmic scale, as shown in the rate key at the left. The numbers on each branch represent the time of divergence from the most recent common ancestor. |

| 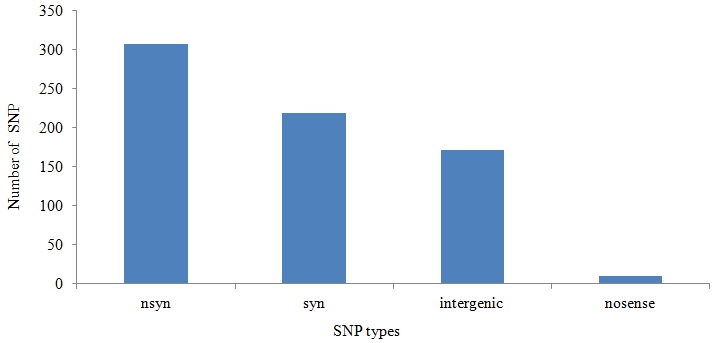 |
| --- |
| **Supplementary Figure S5.** Distribution of different SNP type |

| **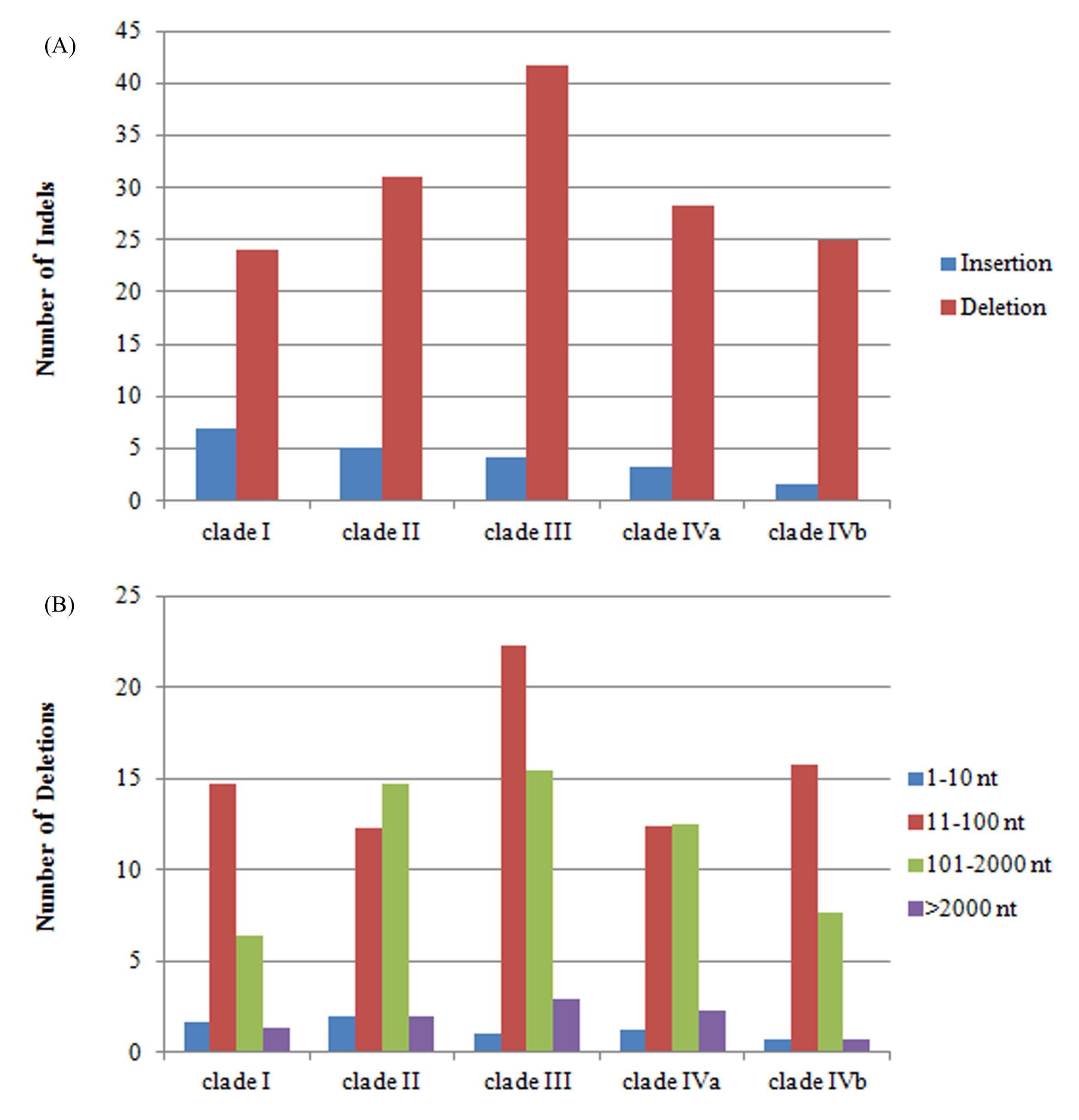** |
| --- |
| **Supplementary Figure S6.** Ongoing reductive evolution in the *B. pertussis* population. (A) The mean number of insertions/deletions from different clades. (B) The distribution of deletions in the different clade according to their nucleotide size Data represent the mean number of all isolates from each clade. |

| 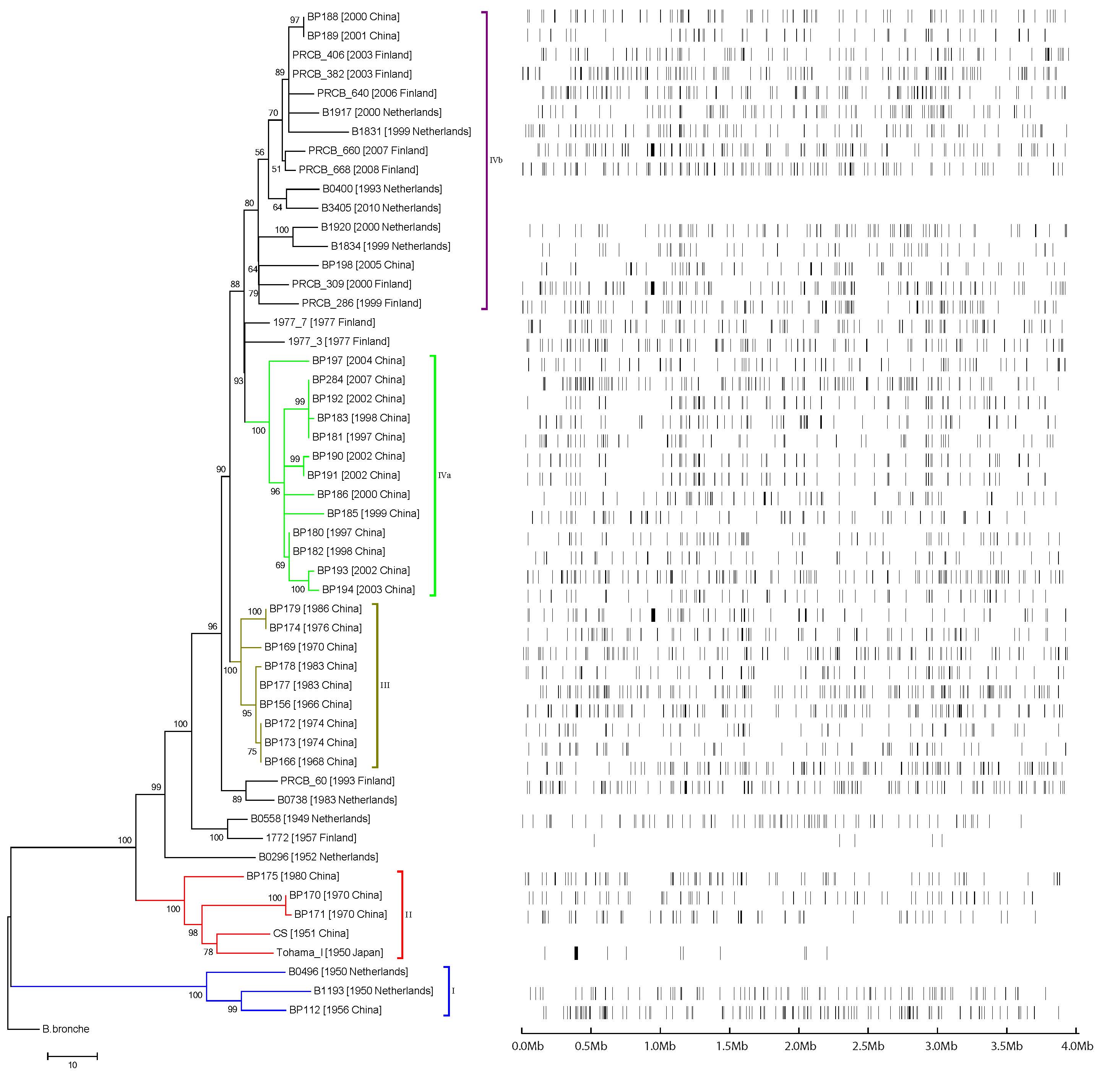 |
| --- |
| **Supplementary Figure S7**. The accumulation of deletions in *B. pertussis*. The deletions were mapped to the primary branch of the phylogenetic tree presented in Figure 1. Deletion data of five isolates (B0400, B3405, B0738, B0296, and B0496) from Netherlands was not included. |
